# Genomic Characterization of Methicillin-Susceptible *Staphylococcus aureus* Carriage in Patients on Home Parenteral Nutrition and Their Caregivers

**DOI:** 10.1093/cid/ciad721

**Published:** 2023-11-27

**Authors:** Michelle Gompelman, Ingrid J M van Weerdenburg, Guus T J Wezendonk, Jordy P M Coolen, Reinier P Akkermans, Chantal P Rovers, Heiman F L Wertheim, Geert J A Wanten

**Affiliations:** Intestinal Failure Unit, Department of Gastroenterology and Hepatology, Radboud University Medical Center, Nijmegen, The Netherlands; Department of Medical Microbiology and Radboudumc Center for Infectious Diseases, Radboud University Medical Center, Nijmegen, The Netherlands; Intestinal Failure Unit, Department of Gastroenterology and Hepatology, Radboud University Medical Center, Nijmegen, The Netherlands; Department of Medical Microbiology and Radboudumc Center for Infectious Diseases, Radboud University Medical Center, Nijmegen, The Netherlands; Radboud Institute for Health Sciences, IQ Healthcare, Radboud University Medical Centre, Nijmegen, The Netherlands; Department of Primary and Community Care, Radboud University Medical Centre, Nijmegen, The Netherlands; Department of Internal Medicine, Division of Infectious Diseases, Radboud University Medical Center, Nijmegen, The Netherlands; Department of Medical Microbiology and Radboudumc Center for Infectious Diseases, Radboud University Medical Center, Nijmegen, The Netherlands; Intestinal Failure Unit, Department of Gastroenterology and Hepatology, Radboud University Medical Center, Nijmegen, The Netherlands

**Keywords:** *Staphylococcus aureus* carriage, home parenteral nutrition, transmission, whole-genome sequencing, decolonization treatment

## Abstract

In this prospective study, patients on home parenteral nutrition were twice as likely to be colonized with *Staphylococcus aureus* if their caregivers were carriers. Among *S. aureus*-positive patients and their caregivers, molecular analysis showed 68% genetically related strains. Despite decolonization, genetically related strains reappeared in 70% of patients.

*Staphylococcus aureus* carriage is a well-defined risk factor for acquiring *S. aureus* infections [1]. Approximately 19% of the patients with *S. aureus* carriage and a long-term central venous access device (CVAD), such as patients on home parenteral nutrition (HPN), develop an *S. aureus* infection yearly [2]. Close contact is a notable contributor to the spread of *S. aureus*; however, transmission from the environment where bacteria can survive for weeks is also reported [1]. A systematic review showed a transmission risk of ±40% within households [3], indicating that close contacts may serve as an external reservoir for *S. aureus* (re)colonization and infection. Many studies used non–whole-genome sequencing (non-WGS) based genotyping to confirm that people of the same household carry genetically related *S. aureus* strains [3, 4]. These methods lack resolution to confirm transmission between patients and their close contacts. Furthermore, such data in HPN patients are lacking, while most patients depend on caregivers who carry out most of the CVAD handling. We hypothesize that due to close contact and *S. aureus* carriage, transmission, persistent colonization, and CVAD-related infections including *S. aureus* bacteremia (SAB) are more likely. Genetic variations between carriage strains were explored using WGS.

## METHODS

This prospective, observational study was conducted as part of a randomized multicenter study (CARRIER trial: Long-term *Staphylococcus aureus* decolonization in patients on home parenteral nutrition) [5]. In short, *S. aureus* screening of adult HPN patients and their caregivers (close contacts who assist in CVAD care) was performed as part of the eligibility screening (2017–2021). Exclusion criteria were HPN discontinuation, hospitalization, antibiotic treatment, and multiple caregivers (eg, home care). If patients underwent decolonization treatment, additional cultures were collected every 3 months for 1 year. Two types of treatments were administered (Supplementary Figure 1): continuous suppression (CS), which is a monthly repeated chronic topical antimicrobial treatment, and search and destroy (SD), which is a short systemic and topical antimicrobial treatment (only repeated in case of recolonization).

Caregivers did not receive decolonization treatment. Ethical approval was obtained from each participating hospital.

Primary outcomes were the association and genetic relatedness of *S. aureus* carriage among HPN patients and their caregivers. We also explored the genetic relatedness of *S. aureus* strains of patients who underwent decolonization treatment over time. Strains of SAB events that took place during the study period were compared to carriage strains.

Sample collection and DNA extraction for WGS analysis are described in Supplementary Appendix 1. Bioinformatic tools were used for complete genome analysis and to detect genetic variation at the single-nucleotide polymorphism (SNP) level. Core SNP differences between every genome were calculated; a cutoff of ≤30 SNPs was used to define genetically related strains (Supplementary Appendix 2). Phylogeny and visualization are described in Supplementary Appendix 3. Genomes were linked to epidemiological data for analysis in SPSS (Supplementary Appendix 4). Binary logistic regression analysis assessed the association of *S. aureus* carriage in HPN patients and their caregivers, expressed in odds ratios (ORs) and 95% confidence intervals (CIs). Possible confounders (age, gender, previous *S. aureus* infections [<2 years], presence of [gastro]enterostomy, and CVAD insertion site [1, 6]) were included as covariates.

## RESULTS

In total, 412 persons were screened for eligibility, and 241 HPN patients were included; 127 (53%) had a caregiver (Supplementary Figure 2). A total of 63 HPN patients received decolonization treatment (SD, 32; CS, 31). Study population characteristics are presented in Supplementary Table 1. In total, 46% (112 of 241) of HPN patients and 48% (61 of 127) of cultured caregivers were *S. aureus* carriers. All isolates concerned methicillin-susceptible *S. aureus*. The most common sites of colonization in HPN patients and their caregivers were the nose (77% and 87%, respectively) and throat (49% and 62%, respectively). HPN patients were more likely to be *S. aureus* carriers when their caregiver was also a carrier (OR, 2.32; 95% CI, 1.13–4.76; *P* = .02; Supplementary Table 2).

In 38 cases, both the HPN patient and their caregiver were colonized with *S. aureus*; 68% of their strains were genetically related (26 of 38, 3 missing). No correlation was found between SNP differences (range, 0–8125 SNPs) and culture collection time (range, 0–1048 days) for both HPN patients’ strain and their caregivers’ strain (*r =* 0.265; *P* = .2; Supplementary Figure 3). The phylogeny of the study cohort is illustrated in Supplementary Figures 4 and 5.

**Figure 1.**
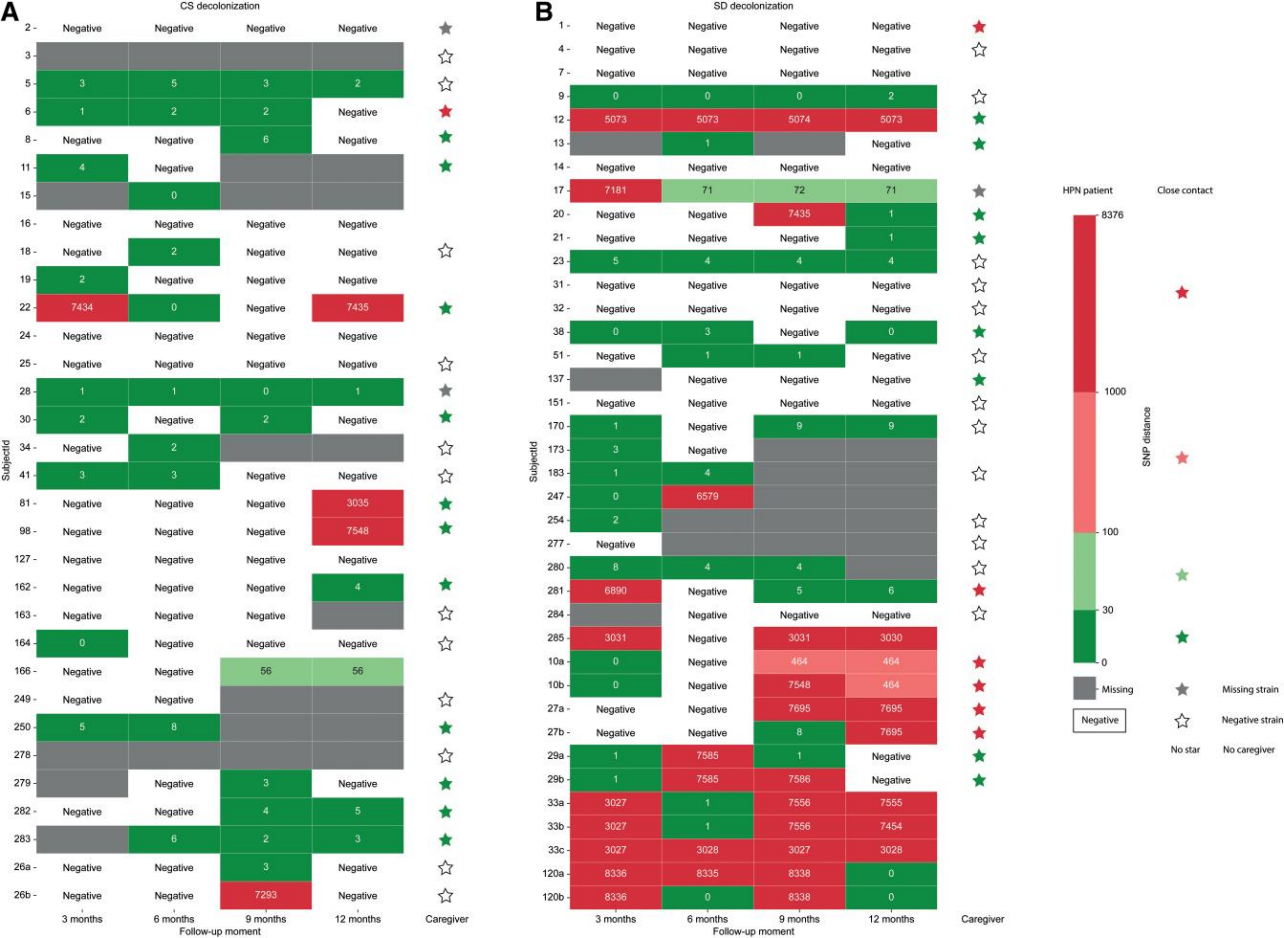
Heat map showing the SNP distance between follow-up cultures of the HPN patients who received decolonization treatment (n = 63) related to baseline colonization culture. Gray shading corresponds to a missing sample (due to no culture taken or patient dropped out of study). Lowercase letters reflect genetically different isolates found on the same follow-up moment. *A*, Patients who received CS decolonization treatment. *B*, Patients who received SD decolonization treatment. Abbreviations: CS, continuous suppression; HPN, home parenteral nutrition; SD, search and destroy; SNP, single-nucleotide polymorphism; SubjectId, participant number.

The temporal pattern of *S. aureus* carriage and genetic relatedness in HPN patients who received decolonization treatment (n = 63) [5] is shown in Figure 1. During the 1-year follow-up, *S. aureus* carriage recolonization occurred in 70% (44 of 63) of these participants. In 28 patients, all recultured isolates were genetically related to the baseline strain; 5 patients had genetically different *S. aureus* strains at follow-up; and 11 patients had multiple *S. aureus* strains (genetically related and different strains) isolated during the year (Figure 1, Supplementary Figure 6). Details of Sequence Types can be found in Supplementary Appendix 5 and Supplementary Table 3.

Seven HPN patients suffered from SAB during the study period (Supplementary Table 4); 71% (5/7) of the patients had positive colonization cultures; all were genetically related to the SAB strain. One patient had 2 SAB episodes; 1 strain was genetically related (5 SNPs) to the colonization strain, while the other strain was genetically different (8137 SNPs).

## DISCUSSION

Pathogen transmission between patients and their close contacts may be an overlooked issue. This notion is supported by our WGS analysis of *S. aureus* carriage isolates from HPN patients and their caregivers. We found genetic relatedness, evidenced by a ≤30 SNP distance in 68% of these strains.

Our findings show that *S. aureus* transmission occurs in the HPN population, which agrees with current literature [7]. Moreover, studies of households of patients with community-acquired methicillin-resistant *S. aureus* (MRSA) infections suggest that the home environment, including close contacts and environmental surfaces, is a critical MRSA reservoir [8]. MRSA eradication guidelines only recommend considering eradication of the household when *S. aureus* decolonization failure occurs [9]. Recently, we found that *S. aureus* eradication failure, next to possession of a (gastro)enterostomy, was associated with an *S. aureus-*positive caregiver [5]. Compliance and type of decolonization (CS or SD) did not influence the risk of decolonization failure. We showed that the successful eradication rate in SD group participants decreased from 66% at start to 41% at 3 months, suggesting recolonization by external factors. Taken together, it seems relevant to screen and, preferably, directly eradicate caregivers as well.

A SNP threshold of ≤30 defined genetic relatedness; however, the literature is not consistent in this aspect. Coll et al [10] calculated a SNP cutoff by adding the 95th percentile value from the modeled cloud of diversity distribution to the number of SNPs suspected to accumulate over time in the source and recipient. Our 95th percentile of within-host diversity was 7 core-genome SNPs, which was lower than Coll et al reported. Their literature review found substitution rates that ranged from 2 to 10 SNPs per genome per year. Applying a lower threshold of ≤17 SNPs to our data reduced genetic relatedness between the HPN patient and the caregiver to 63% (n = 2 less).

From the temporal pattern of *S. aureus* isolates in HPN patients receiving decolonization treatment, it is clear that some patients carry multiple strains. The simultaneous carriage of more than 1 *S. aureus* strain is important since it can be a reservoir for horizontal gene transfer, transfer of antibiotic resistance, and virulence factors between co-colonizing strains [11]. This could contribute to the pathogenicity of the particular isolate and the risk for severe infections or decolonization failure.

We found that HPN patients with *S. aureus* bacteremia carried *S. aureus* on their body in 70% of the cases, with genetically related strains in all patients. This agrees with a study comparing nasal strains during SAB [12].

By including a high percentage of cultured HPN patients, this study represents a reliable display of the Dutch HPN population. To lower the risk of false-negative carriage findings, we cultured extranasal body parts. The rather long follow-up duration enabled us to generate a detailed overview of *S. aureus* carriage and potential time-related effects. Use of WGS provided more resolution than non-WGS methods to study genetic relatedness since the entire genome was scrutinized. A limitation of our study, however, is that we did not obtain all cultures from HPN patients and their caregivers simultaneously and repeatedly. The result could be an underrepresentation of genetic relatedness between these isolates. Yet, no correlation was seen between the difference in screening date and SNP difference, illustrating that certain *S. aureus* strains are carried over a long period. Second, sample sizes for certain subanalyses were relatively small.

In conclusion, we demonstrate that *S. aureus* carriage among HPN patients is more likely if their caregiver also carries this pathogen. Molecular analysis showed that most patients carry the same *S. aureus* strain as their caregiver. When HPN patients developed *S. aureus* bacteremia, carriage and bacteremia strains were genetically related. Following decolonization, genetically related strains reappeared in most patients after 1 year. Our findings suggest an important role of caregivers in *S. aureus* transmission in HPN patients and the potential value in eradicating *S. aureus* in caregivers as well as patients.

## Supplementary Data

Supplementary materials are available at *Clinical Infectious Diseases* online. Consisting of data provided by the authors to benefit the reader, the posted materials are not copyedited and are the sole responsibility of the authors, so questions or comments should be addressed to the corresponding author.

## Supplementary Material

ciad721_Supplementary_Data

## Notes

***Financial support*.** This work was supported by a government grant (ZonMw; grant number 848015009).
