# Supplementary material for: Genomic Characterization of Methicillin-Susceptible Staphylococcus aureus Carriage in Patients on Home Parenteral Nutrition and Their Caregivers
Source: Clin Infect Dis. 2023 Nov 27;78(5):1285–8. doi: 10.1093/cid/ciad721 (PMC11093653; doi:10.1093/cid/ciad721)
Supplement: ciad721_Supplementary_Data [file ciad721_supplementary_data.docx]

**Appendix A. Supplementary data**

**Genomic characterization of methicillin-susceptible *Staphylococcus aureus* carriage in patients on home parenteral nutrition and their caregivers**

Michelle Gompelman^1^, Ingrid J.M. van Weerdenburg^2^, Guus T.J. Wezendonk^1^, Jordy P.M. Coolen^2^ , Reinier P. Akkermans^3,4^, Chantal P. Rovers^5^, Heiman F. L. Wertheim^2^, Geert J.A. Wanten^1^

1 Intestinal Failure Unit, Department of Gastroenterology and Hepatology, Radboud University Medical Center, Nijmegen, the Netherlands.

2 Department of Medical Microbiology and Radboudumc Center for Infectious Diseases, Radboud University Medical Center, Nijmegen, the Netherlands.

3 Radboud Institute for Health Sciences, IQ healthcare, Radboud university medical centre, Nijmegen, The Netherlands

4 Department of Primary and Community Care, Radboud university medical centre, Nijmegen, The Netherlands

5 Department of Internal Medicine, Division of Infectious Diseases, Radboud University Medical Center, Nijmegen, the Netherlands.

### Table of contents:

| **Type** | **Description** | **Page number** |
| --- | --- | --- |
| **Appendix S1** | Collection of *S. aureus* samples and WGS analysis | 2 |
| **Appendix S2** | Method of whole-genome sequencing analysis | 2 |
| **Appendix S3** | Phylogeny and visualization | 3 |
| **Appendix S4** | Statistical analyses | 3 |
| **Appendix S5** | Additional molecular analysis results | 4 |
| **Figure S1** | Flow chart *S. aureus* decolonization treatments | 5 |
| **Figure S2** | Flow chart showing the study cohort and genomic analysis and reasons for exclusion. | 6 |
| **Figure S3** | Heatmap of the SNP distances and culture date differences. | 7 |
| **Figure S4** | Phylogenetic tree based on all 408 *S. aureus* isolates of study cohort | 8 |
| **Figure S5** | Phylogenetic tree of study cohort with the HPN patient and their corresponding caregiver highlighted in the same color. | 9 |
| **Figure S6** | Visualization of temporal pattern of *S. aureus* carriage of HPN patients who received decolonization treatment. | 10 |
| **Table S1** | Study population characteristics. | 11 |
| **Table S2** | Crosstabulation of *S. aureus* carriage among HPN patients and their caregivers. | 12 |
| **Table S3** | Allelic profile and SNP mutation for the 28 genomes where the loci did not match perfectly to any known *S. aureus* MLST alleles. | 13 |
| **Table S4** | SNP distance between SAB strain and the colonization culture of the HPN patient. | 14 |

**Appendix S1.** Collection of *S. aureus* samples and WGS analysis

*S. aureus* cultures were collected from the nares, throat, perineum, gastro(entero)stomy (when present), and CVAD insertion site (HPN patients only). *S. aureus* screening of caregivers was performed preferably within three months of the culture date of the corresponding HPN patient. Screening swabs were placed in 1 ml amies transport medium (COPAN, Bréscia, Italy) and mixed with a vortex mixer at the microbiological department of the Radboudumc. The swab was plated on a Columbia blood agar (BD, Heidelberg, Germany) and incubated for two days at 36˚C CO2. Identification of *S. aureus* in 2018 and 2019 was performed using the coagulase test [1,2]. In 2019 the Radboudumc transitioned to *S. aureus* identification with Matrix-Assisted Laser Desorption Ionization Time Of Flight Mass Spectroscopy (MALDI-TOF MS, Bruker Daltonics, Bremen, Germany) [3]. All positive *S. aureus* cultures were stored in glycerol-containing liquid media at -80˚C. Additionally, pure cultures on Colombia blood agar of *S. aureus* were subjected for WGS.

DNA extraction for WGS analysis was performed using Instagene Matrix (Bio-rad, Hercules, CA, USA). DNA sequencing was performed on a G400 sequencer (BGI Genomics, Denmark) using the DNA nanoball sequencing (DNBSEQ) technology [4].

**References**

1. MacFaddin JF. Biochemical Tests for Identification of Medical Bacteria. 3rd Edition. 2000: Lippincott Williams & Wilkins, Philadelphia.
2. Leber AL. Clinical Microbiology Procedures Handbook, 3 Volume Set. 2016: Wiley.
3. Singhal N, Kumar M, Kanaujia PK, Virdi JS. MALDI-TOF mass spectrometry: an emerging technology for microbial identification and diagnosis. Front Microbiol. 2015 Aug 5;6:791. doi: 10.3389/fmicb.2015.00791. PMID: 26300860; PMCID: PMC4525378.
4. Drmanac R, Sparks AB, Callow MJ, Halpern AL, Burns NL, Kermani BG, et al. Human genome sequencing using unchained base reads on self-assembling DNA nanoarrays. Science. 2010 Jan 1;327(5961):78-81. doi: 10.1126/science.1181498. Epub 2009 Nov 5. PMID: 19892942.

**Appendix S2. Method of whole-genome sequencing analysis**

For genome assembly and species identification, paired-end 150-bp FASTQ files were subjected to the pipeline Bactopia version 1.6.5 [1]. Multilocus sequencing typing (MLST) of *S. aureus* was performed [16]. Genomes with poor data quality or a coverage less than 30x were excluded from further analysis. The *S. aureus* reference strain NCTC 8325 (Accession NC_007795.1, available in NCBI) and study *S. aureus* genomes were compared using the core genome SNPs as identified by kSNP3 (version 3.1.2) [2].

Recent literature proposed a SNP cutoff of > 15 SNP after six months to exclude transmission [3]. Since our trial had a follow-up duration of 12 months, we defined genetic relatedness in case single nucleotide polymorphism (SNP) difference was ≤ 30. SNP distances between the strains of different participants or within one participant was explored to investigate if the genomes were genetically related. Otherwise, the strains were considered as genetically different strains.

**References**

1. Petit RA 3rd, Read TD. Bactopia: a Flexible Pipeline for Complete Analysis of Bacterial Genomes. mSystems. 2020 Aug 4;5(4):e00190-20. doi: 10.1128/mSystems.00190-20. PMID: 32753501; PMCID: PMC7406220.
2. Maiden MC. Multilocus sequence typing of bacteria. Annu Rev Microbiol. 2006;60:561-588. doi:10.1146/annurev.micro.59.030804.121325.
3. Coll F, Raven KE, Knight GM, Blane B, Harrison EM, Leek D, Enoch DA, Brown NM, Parkhill J, Peacock SJ. Definition of a genetic relatedness cutoff to exclude recent transmission of methicillin-resistant Staphylococcus aureus: a genomic epidemiology analysis. Lancet Microbe. 2020 Dec;1(8):e328-e335. doi: 10.1016/S2666-5247(20)30149-X. PMID: 33313577; PMCID: PMC7721685

**Appendix S3. Phylogeny and visualization**

Phylogeny was inferred using the core SNP consensus parsimony. Phylogenetic trees were made based on 33.672 core SNPs of the 408 *S. aureus* isolates from the study cohort (Figures S4 and S5). Final phylogeny visualizations were performed in Microreact [1]. Visualization tool Cytoscape was used to create a network graph [2]. Python version 3.10 was used to create heatmaps to illustrate SNP distances (Python Software Foundation).

**References**

1. Argimón S, Abudahab K, Goater RJE, et al. Microreact: visualizing and sharing data for genomic epidemiology and phylogeography. Microb Genom. 2016;2(11):e000093. Published 2016 Nov 30. doi:10.1099/mgen.0.000093.
2. Shannon P, Markiel A, Ozier O, Baliga NS, Wang JT, Ramage D, Amin N, Schwikowski B, Ideker T. Cytoscape: a software environment for integrated models of biomolecular interaction networks. Genome Research 2003 Nov; 13(11):2498-504.

**Appendix S4. Statistical analyses**

Patient characteristics are described as mean and standard deviation for continuous variables and numbers with percentages for categorical variables. In case of missing data, the actual numbers are depicted in the respective tables. We performed univariate binary logistic regression analysis to assess the association of *S. aureus* carriage in HPN patients and their caregivers, expressed in Odds Ratios (OR) and 95% confidence intervals. Wald test was used to test the effect of the factors in the logistic model. Potential risk factors and/or confounders for *S. aureus* carriage were identified by literature: age, gender, previous *S. aureus* infections within 2 years, presence of (gastro)enterostomy, and CVAD insertion site [1-3]. These confounders were added to the binary logistic regression model to analyze the effect on the uncorrected univariate OR. A change of more than 10% on the estimated effect (expressed in logit) from the univariate analysis was considered clinically relevant. A value of *p* <0.05 was considered statistically significant for all analyses based on two-sided testing. Pearson’s correlation was performed to control for a potential relationship between SNP difference and *S. aureus* screenings date difference. To compare *S. aureus* cultures of HPN patients and those of the corresponding caregiver, the HPN patients’ culture most close to the screening date of the caregiver was used for genetic analysis. This study considered > 90 days between dates of collected cultures from HPN patients and their caregivers to be a substantial screening difference. All statistical analysis was performed in SPSS version 27.0 (IBM, Armonk, NY, USA).

**References**

1. Wertheim HF, Melles DC, Vos MC, van Leeuwen W, van Belkum A, Verbrugh HA, Nouwen JL. The role of nasal carriage in Staphylococcus aureus infections. Lancet Infect Dis. 2005 Dec;5(12):751-62. doi: 10.1016/S1473-3099(05)70295-4. PMID: 16310147.
2. Gompelman M, Wezendonk GTJ, Wouters Y, et al. Randomized clinical trial: Long-term *Staphylococcus aureus* decolonization in patients on home parenteral nutrition [published online ahead of print, 2023 Mar 17]. Clin Nutr. 2023;42(5):706-716. doi:10.1016/j.clnu.2023.03.010
3. Ammerlaan HS, Kluytmans JA, Berkhout H, Buiting A, de Brauwer EI, van den Broek PJ, van Gelderen P, Leenders SA, Ott A, Richter C, Spanjaard L, Spijkerman IJ, van Tiel FH, Voorn GP, Wulf MW, van Zeijl J, Troelstra A, Bonten MJ; Eradication of carriage with methicillin-resistant Staphylococcus aureus: determinants of treatment failure. J Antimicrob Chemother. 2011 Oct;66(10):2418-24. doi: 10.1093/jac/dkr250. Epub 2011 Jun 30. PMID: 21719471.

**Appendix S5. Additional molecular analysis results**

*ST type - overall*

Thirty-four STs and 8 clonal complexes (CC) were found among the genomes in this study with CC5/ST5 as the most prevalent one (Figure S4). Five genomes were predicted to be in a novel ST. These genomes belonged to the same patient and his caregiver and showed the same identical allelic profile. Twenty-eight genomes had one or two loci which did not match perfectly to any known *S. aureus* MLST alleles (Table S3). Grouping these genomes per patient, the same SNP mutation in the loci was found (≤14 SNP difference).

*ST type - Multiple body sites*

Each participant (HPN patient or caregiver) was sampled on multiple body sites per timepoint (screening or follow-up). Genomes found on different body sites with an equal ST type, showed a SNP distance between 0 and 14 SNPs. Remarkably, two genomes (HPN 0; CC 2) showed a higher SNP distance while having the same allelic profile (ST188: 33 SNPs, ST8: 72 SNPs). Therefore, we considered those genomes as genetically different. Thirteen genomes (HPN 9; CC 2) showed a different allelic profile (ST type). SNP distance varied between 106 and 8355 for those isolates and were considered as genetically different. Likely, these participants had >1 *S. aureus* strains cultured on their body sites.

*ST type - HPN patient and his caregiver*

A different ST type was found in one comparison of a HPN patient (ST22) and his caregiver (not defined ST); MLST analysis, however, showed the same allelic profile as ST22 for the non-defined genome, except for a A471G mutation in the *pta* gene.

*ST type - S. aureus recolonization*

63% (40/63) of the HPN patients had recolonization strains with the same ST type as their baseline colonization strain. All SNP distances were less than 13 SNPs, except for one. One recolonization strain differed 56 SNPs with the baseline strain, while ST type was 8 for both isolates.

**Figure S1.** Flow chart *S. aureus* decolonization treatments.


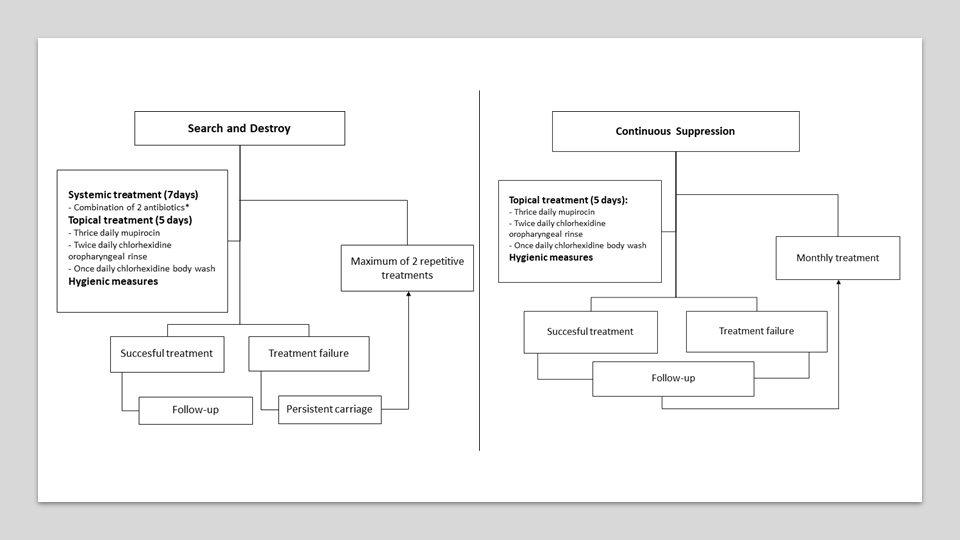


*Systemic antibiotic options included: teicoplanin (IV), clindamycin (IV/PO), ciprofloxacin (IV/PO), co-trimoxazole (IV/PO), clarithromycin (PO), fusidic acid (PO), doxycycline (IV/PO) and rifampicin (IV/PO).

**Figure S2.** Flow chart showing the study cohort and genomic analysis and reasons for exclusion.


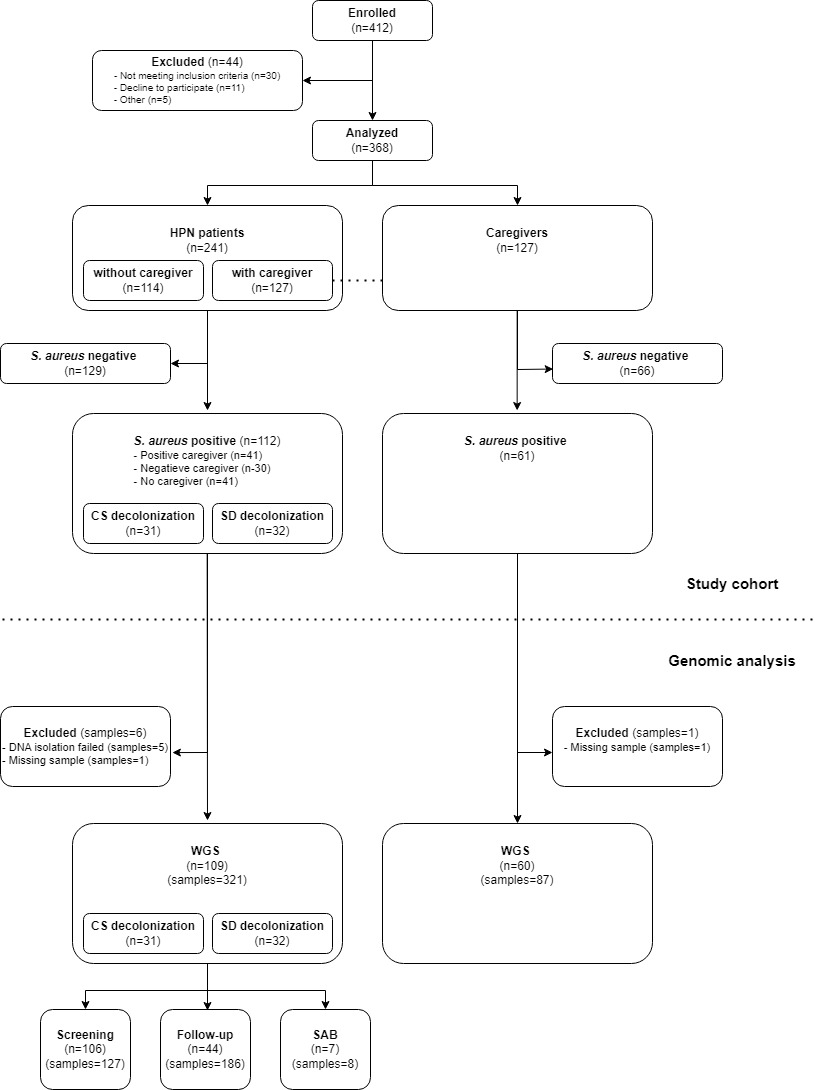


**Abbreviations**: CS: Continuous Suppression decolonization treatment; HPN: home parental nutrition; SAB: *S. aureus* bacteremia; SD: Search and Destroy decolonization treatment; WGS: whole-genome sequencing.

**Figure S3**. Heatmap of relation between difference in culture collection dates and SNP distances (between strain of HPN patient and strain of caregiver).


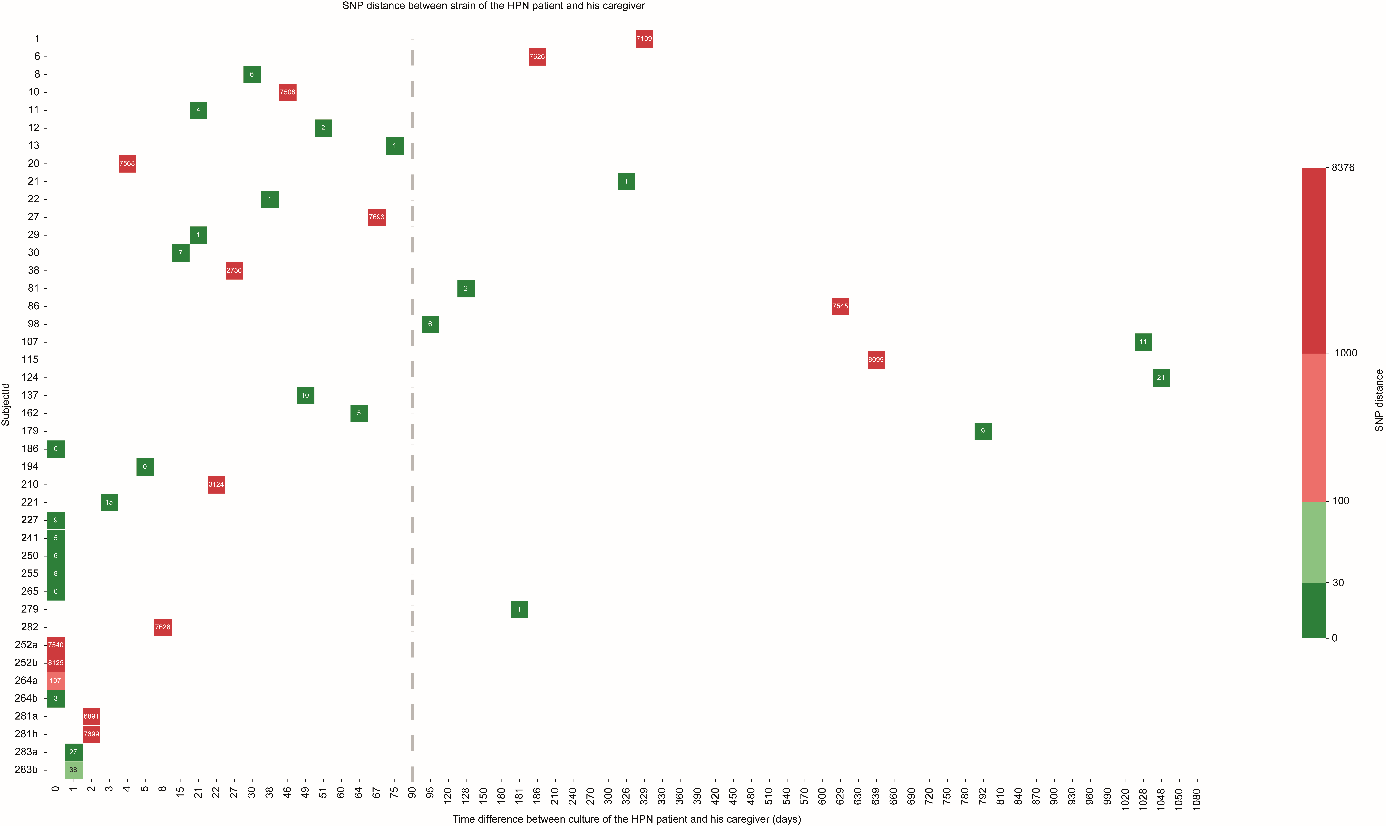


Heatmap showing the SNP distance between the isolate of the HPN patient with their caregiver related to the time difference (in days) between both isolates. X-axis: not linear representation of the time difference (days) between both cultures. Y-axis: SubjectId. Dark green corresponds to genetically related strains (SNP distance ≤30). Light green, light red and dark red corresponds to genetically different strains. The vertical gray dashed line corresponds to a time difference of 90 days between both isolates. A time difference of 90 days or more was considered to be a large screening difference.
**Abbreviations**: HPN: home parental nutrition; SNP: single nucleotide polymorphisms.

**Figure S4.** Phylogenetic tree generated from core genome SNPs of all 408 *S. aureus* isolates (109 HPN patients; 60 caregivers). STs are distinguished by a specific color.


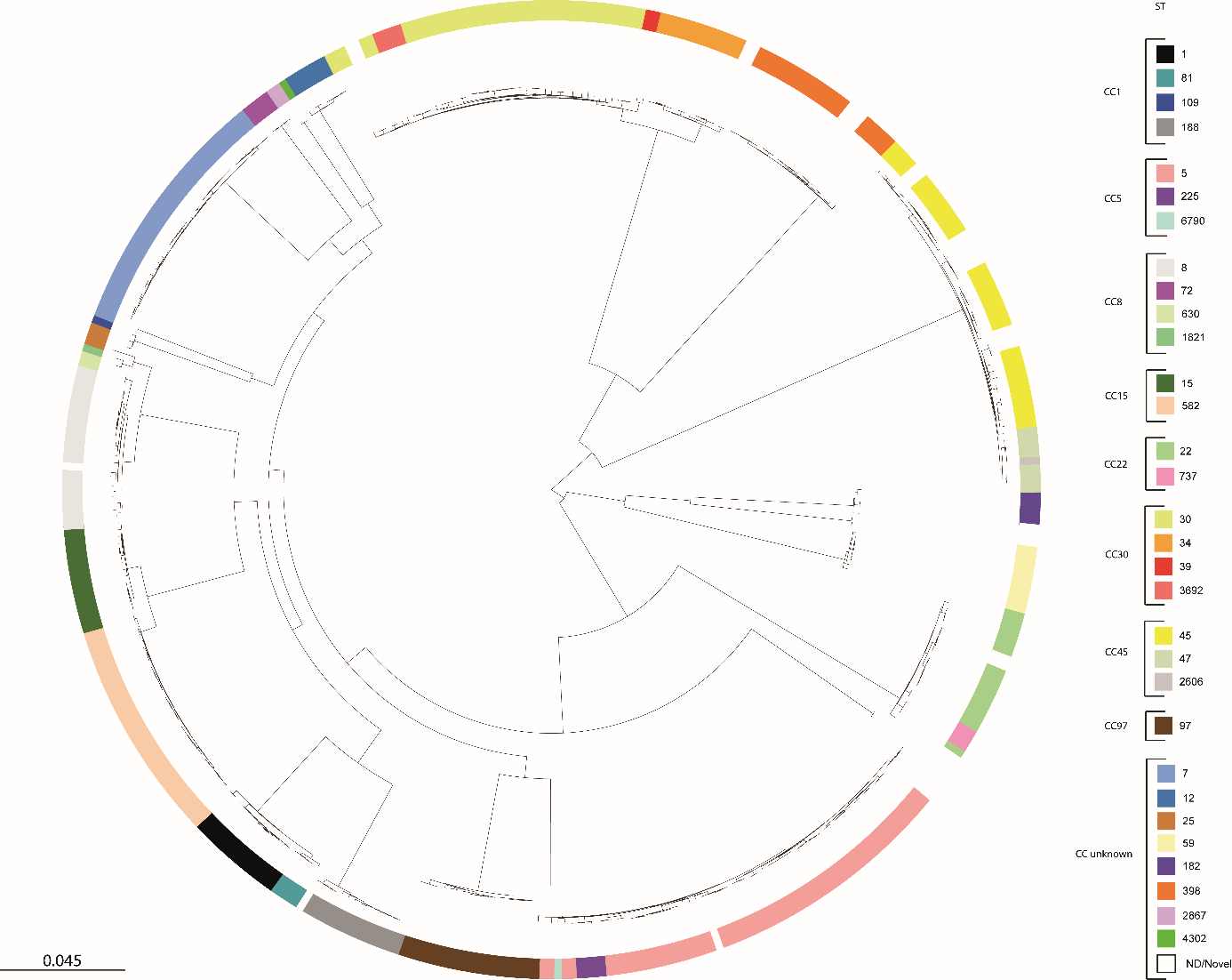


**Abbreviations**: CC: clonal complex; HPN: home parental nutrition; SNP: single nucleotide polymorphisms; ST: sequence type.

**Figure S5.** Phylogenetic tree based on all 408 *S. aureus* isolates, where the cohort of HPN patient (square symbol) and their corresponding caregiver (star symbol) are highlighted in the same color.


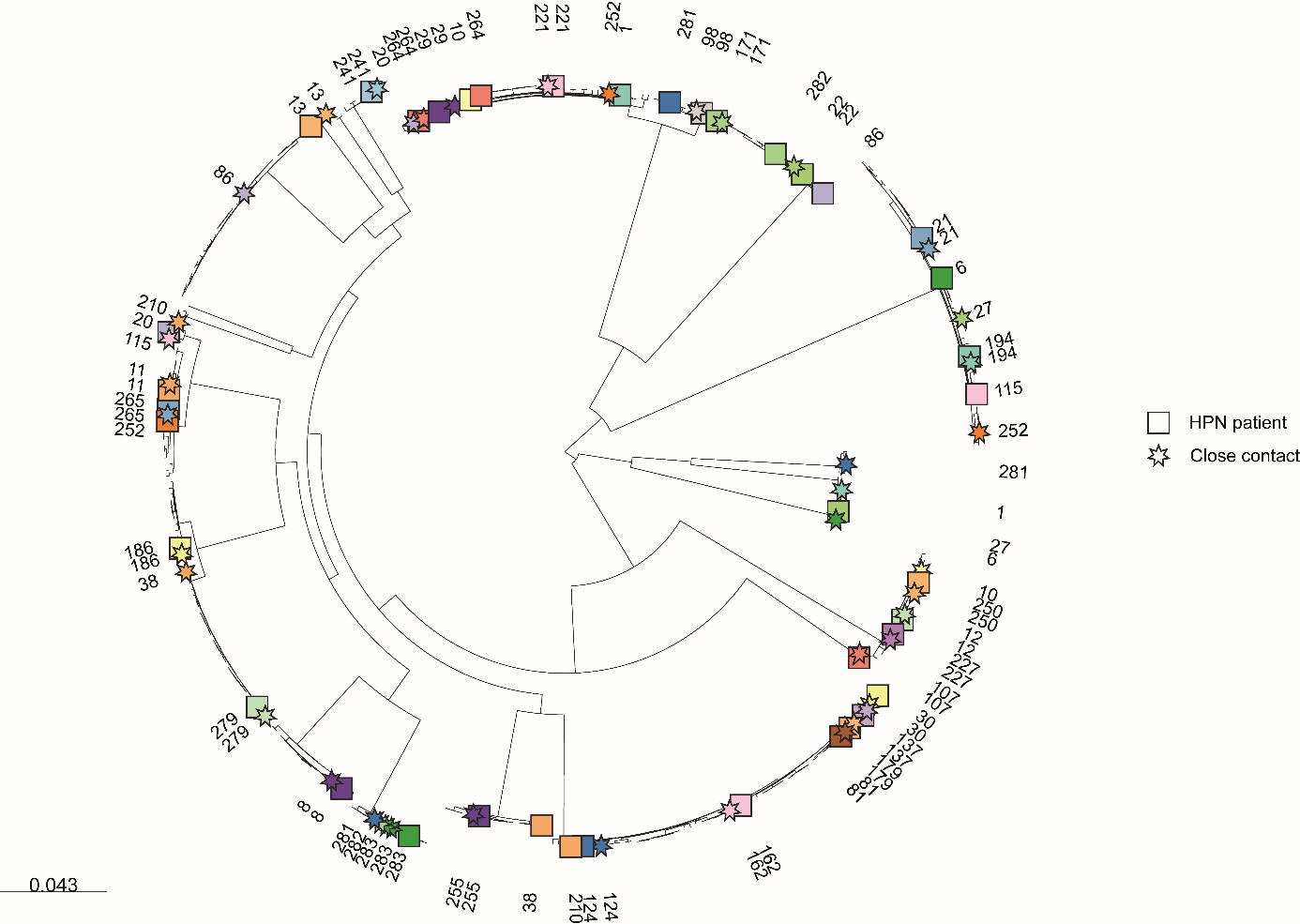


**Abbreviations**: HPN: home parental nutrition**.**

**Figure S6**. Visualization of temporal pattern of *S. aureus* carriage of HPN patients with genetic different strains during follow-up, while receiving decolonization treatment.


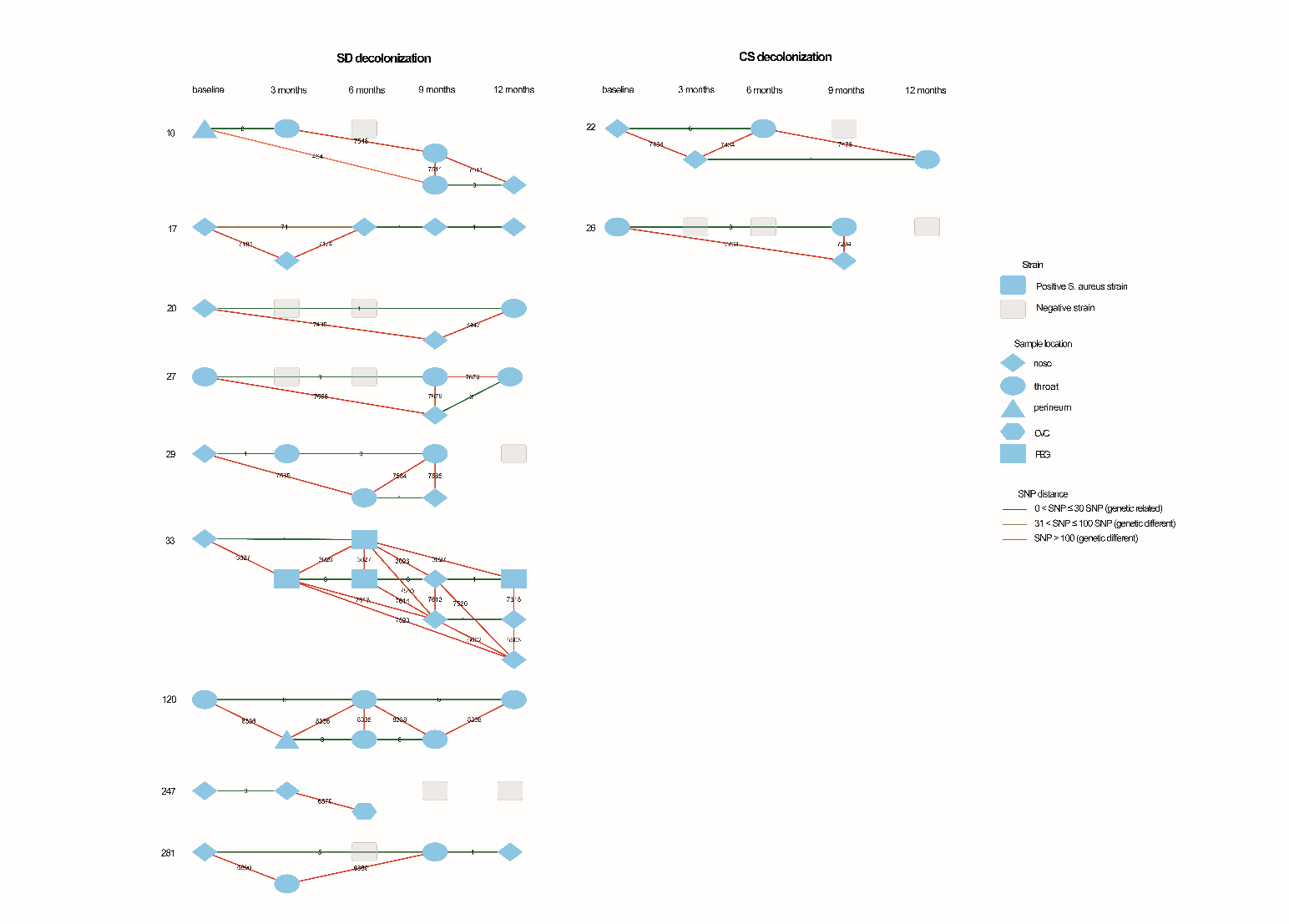


Only HPN patients with genetically different strains are visualized. The green line reflects a SNP distance ≤30 SNPs between those samples (*e.g*. genetically related). A red line reflects a SNP distance of >30. **Abbreviations**: CS: Continuous Suppression; CVC: Central venous catheter; HPN: home parental nutrition**;** PEG: percutaneous endoscopic gastro(entero)stomy; SD: Search and Destroy; SNP: single nucleotide polymorphisms.

**Table S1.** Study population characteristics. **A**. Characteristics of the cultured HPN patients and their caregivers. **B**. Characteristics of the cultured HPN patients and their caregivers with confirmed *S. aureus* carriage. **C.** CVAD type and site of insertion (HPN patients only).

**A.**

| Category |  | HPN patients (N=127) | Caregivers (N=127) |
| --- | --- | --- | --- |
| Age in years, mean (range) |  | 54 (19 – 78) | 58 (25 – 85) |
| Gender | Male | 40 (31%) | 86 (68%) |
| Type of caregiver | Partner |  | 117 (92%) |
|  | Family member |  | 10 (8%) |
| *S. aureus* carriage | Yes | 71 (56%)* | 61 (48%) |

**B.**

| Category |  | HPN *S. aureus* carriage  (N=71) | Caregivers *S. aureus* carriage  (N=61) |
| --- | --- | --- | --- |
| Location *S. aureus* carriage | Nose | 55 (77%) | 53 (87%) |
|  | Throat | 35 (49%) | 38 (62%) |
|  | Perineum | 21 (30%) | 28 (46%) |
|  | CVAD | 20 (28%) | NA |
|  | Gastro(entero)stomy (n=21) | 6 (29%) | NA |
| *S. aureus* carriage locations | 1 | 30 (42%) | 21 (34%) |
|  | 2 | 22 (31%) | 22 (36%) |
|  | >2 | 19 (27%) | 18 (30%) |
| Extra nasal *S. aureus* carriage | Yes | 54 (76%) | 38 (62%) |
| *S. aureus* infections < 2 years^^^ | Yes | 13 (18%) | Unknown |

**C.**

| Category | HPN patients (N=127) |
| --- | --- |
| CVAD type |  |
| Tunneled CVC | 81 (64%) |
| Non-Tunneled CVC | 7 (6%) |
| Implanted port | 24 (19%) |
| Arteriovenous fistula | 15 (12%) |
| CVAD insertion site^#^ | N=112 |
| Jugular vein | 90 (80%) |
| Subclavian vein | 10 (9%) |
| Femoral vein | 3 (3%) |
| Other^$^ | 8 (7%) |

*Only HPN patients with *S. aureus* carriage and a cultured caregiver were counted (n=41 excluded). ^All type of infections with a positive *S. aureus* (blood)culture. **^#^**Excluding patients with arteriovenous fistula (n=15). **^$^**Peripherally inserted central lines in brachial, cephalic or basilic veins.  **Abbreviations:** CVAD: central venous access; CVC: central venous catheter; HPN: home parenteral nutrition. NA: not applicable.

**Table S2:** Crosstabulation of *S. aureus* carriage among HPN patients and their caregivers.

|  | | Caregiver | | |
| --- | --- | --- | --- | --- |
|  |  | *S. aureus* positive | *S. aureus* negative | Total |
| HPN patients | *S. aureus* positive | 41 (58%) | 30 (42%) | 71 |
|  | *S. aureus* negative | 20 (36%) | 36 (64%) | 56 |
| Total |  | 61 (48%) | 66 (52%) | 127 |

HPN patients were more likely to be a S. aureus carrier if their caregiver was also a carrier OR 2.32 (95% CI 1.13, 4.76, p = 0.02). Multivariate analysis with possible confounders showed no clinically relevant change on the effect of the univariate ‘S. aureus carriage caregiver’ (adjusted OR 2.16 (0.99 – 4.74). **Abbreviations:** CI: confidence interval; HPN: home parenteral nutrition; OR: Odds ratio.

**Table S3.** Allelic profile and SNP mutation for the 28 genomes where the loci did not match perfectly to any known *S. aureus* MLST alleles.

| arcC | aroE | glpF | gmk | pta | tpi | yqiL | sample | SubjectId | Gene mutation | Gene mutation |
| --- | --- | --- | --- | --- | --- | --- | --- | --- | --- | --- |
| 0 | 5 | 6 | 2 | 7 | 17 | 19 | 1-40 | 18 | arcC6 | C166T |
| 0 | 5 | 6 | 2 | 7 | 17 | 19 | 1-41 | 18 | arcC6 | C166T |
| 0 | 5 | 6 | 2 | 7 | 17 | 19 | 2-44 | 18 | arcC6 | C166T |
| 10 | 14 | 8 | 6 | 10 | 3 | 0 | 1-60 | 21 | yqil2 | G163A |
| 10 | 14 | 8 | 6 | 10 | 3 | 0 | 3-25 | 21 | yqil2 | G163A |
| 10 | 14 | 8 | 6 | 10 | 3 | 0 | 3-26 | 21 | yqil2 | G163A |
| 10 | 14 | 8 | 6 | 10 | 3 | 0 | 3-27 | 21 | yqil2 | G163A |
| 10 | 14 | 8 | 6 | 10 | 3 | 0 | 3-52 | 21 | yqil2 | G163A |
| 3 | 35 | 19 | 2 | 0 | 26 | 39 | 2-25 | 22 | pta20 | A101G |
| 3 | 35 | 19 | 2 | 0 | 26 | 39 | 2-40 | 22 | pta20 | A101G |
| 3 | 35 | 19 | 2 | 0 | 26 | 39 | 3-47 | 22 | pta20 | A101G |
| 10 | 14 | 8 | 6 | 10 | 3 | 0 | 2-37 | 27 | yqil2 | A375G |
| 10 | 14 | 8 | 6 | 10 | 3 | 0 | 3-28 | 27 | yqil2 | A375G |
| 10 | 14 | 8 | 6 | 10 | 3 | 0 | 3-63 | 27 | yqil2 | A375G |
| 3 | 3 | 1 | 185 | 0 | 0 | 3 | 2-67 | 98 | pta4 & pta1 | G448T & A376G |
| 856 | 1 | 14 | 15 | 11 | 0 | 3 | 1-26 | 107 | tpi486 | G195A |
| 856 | 1 | 14 | 15 | 11 | 0 | 3 | 4-71 | 107 | tpi486 | G195A |
| 856 | 1 | 14 | 15 | 11 | 0 | 3 | 6-6 | 107 | tpi486 | G195A |
| 1 | 4 | 1 | 4 | 12 | 0 | 10 | 6-39 | 109 | tpi463 | T63G |
| 1 | 0 | 1 | 9 | 1 | 1 | 1 | 5-24 | 132 | aroE1 | C371T |
| 10 | 14 | 0 | 6 | 10 | 3 | 2 | 2-76 | 139 | glpf8 | G312A |
| 10 | 14 | 0 | 6 | 10 | 3 | 2 | 2-77 | 139 | glpf8 | G312A |
| 8 | 2 | 2 | 2 | 6 | 0 | 2 | 1-12 | 171 | tpi98 | C371T |
| 8 | 2 | 2 | 2 | 6 | 0 | 2 | 5-31 | 171 | tpi98 | C371T |
| 7 | 6 | 1 | 5 | 0 | 8 | 6 | 5-16 | 250 | pta8 | A4716 |
| 7 | 6 | 1 | 5 | 0 | 8 | 6 | 5-70 | 250 | pta8 | A4716 |
| 2 | 2 | 2 | 2 | 0 | 3 | 2 | 5-46 | 264 | pta6 | C421T |
| 2 | 2 | 2 | 2 | 0 | 3 | 2 | 5-47 | 264 | pta6 | C421T |

**Abbreviations**: MLST: Multilocus sequencing typing; SNP: single nucleotide polymorphisms.

**Table S4.** SNP distance between *S. aureus* bacteremia strain and the colonization culture of the HPN patient.

| SubjectId | ST | CC | Time between colonization culture and SAB culture (year; months) | SNP distance with colonization culture |
| --- | --- | --- | --- | --- |
| 6 | 45 | 45 | 0y; 7m | 2 |
| 56 | 45 | 45 | 2y; 3m | 6 |
| 87 | 15 | 15 | 1y; 1m | 12 |
| 97 | 30 | 30 | 0y; 11m | Negative colonization culture |
| 128 | 5 | 5 | 0y; 5m | Negative colonization culture |
| 154 | 47 | 45 | 0y; 9m | 3 |
| 159 | 45 | 45 | 0y; 9m | 8137 |
| 159 | 5 | 5 | 1y; 5m | 5 |

When multiple colonization strains were present, the maximum SNP distance was shown. **Abbreviations:** CC: clonal complex; HPN: home parenteral nutrition; SAB: *S. aureus* bacteremia; SNP: single nucleotide polymorphisms; ST: sequence type.
